# Influenza virus infection is associated with increased risk of death amongst patients hospitalized with confirmed pulmonary tuberculosis in South Africa, 2010–2011

**DOI:** 10.1186/s12879-015-0746-x

**Published:** 2015-01-27

**Authors:** Sibongile Walaza, Stefano Tempia, Halima Dawood, Ebrahim Variava, Jocelyn Moyes, Adam L Cohen, Nicole Wolter, Michelle Groome, Claire von Mollendorf, Kathleen Kahn, Marthi Pretorius, Marietjie Venter, Shabir A Madhi, Cheryl Cohen

**Affiliations:** Centre for Respiratory Disease and Meningitis, National Institute for Communicable Diseases (NICD) of the National Health Laboratory Service (NHLS), Private Bag X4, Sandringham, 2131 Johannesburg, Gauteng South Africa; School of Public Health, Faculty of Health Science, University of the Witwatersrand, Johannesburg, South Africa; US Centres for Disease Control and Prevention, Atlanta, GA USA; US Centres for Disease Control and Prevention, Pretoria, South Africa; Medical Research Council: Respiratory and Meningeal Pathogens Research Unit and Department of Science and Technology/National Research Foundation: Vaccine-Preventable Diseases, University of the Witwatersrand, Johannesburg, South Africa; Pietermaritzburg Metropolitan Hospital Complex, KwaZulu- Natal, South Africa; MRC/Wits Rural Public Health and Health Transition Research Unit (Agincourt), Bushbuckridge, South Africa; Tshepong Hospital, North West Province, South Africa; Faculty of Medicine, University of Witwatersrand, Johannesburg, South Africa; Zoonosis Research Unit, Department of Medical Virology, University of Pretoria, Pretoria, South Africa

**Keywords:** Influenza, Tuberculosis, Co-infection, South Africa

## Abstract

**Background:**

Data on the association between influenza and tuberculosis are limited. We describe the characteristics of patients with laboratory-confirmed tuberculosis, laboratory-confirmed influenza and tuberculosis-influenza co-infection.

**Methods:**

Patients hospitalized with severe respiratory illness (acute and chronic) were enrolled prospectively in four provinces in South Africa. Naso/oropharyngeal specimens were tested for influenza virus by real time reverse transcriptase polymerase chain reaction. Tuberculosis testing was conducted as part of clinical management.

**Results:**

From June 2010 through December 2011, 8032 patients were enrolled and influenza testing was conducted on 7863 (98%). Influenza virus was detected in 765 (10%) patients. Among 2959 patients with tuberculosis and influenza results, 2227 (75%) were negative for both pathogens, 423 (14%) were positive for tuberculosis alone, 275 (9%) were positive for influenza alone and 34 (1%) had influenza and tuberculosis co-infection. On multivariable analysis amongst individuals with symptoms for ≥7 days, tuberculosis influenza co-infection was associated with increased risk of death, (adjusted relative risk ratio (aRRR) (6.1, 95% confidence interval (CI) 1.6-23.4), as compared to tuberculosis only infection. This association was not observed in individuals with symptoms for <7 days (aRRR.0.8, 95% CI 0.1-7.0).

**Conclusion:**

Tuberculosis and influenza co-infection compared to tuberculosis single infection was associated with increased risk of death in individuals with symptoms ≥7 days. The potential public health impact of influenza vaccination among persons with laboratory-confirmed tuberculosis should be explored.

## Background

Despite the availability of efficacious treatment, tuberculosis remains the second most common cause of infectious disease–related deaths worldwide, after human immunodeficiency virus (HIV) and acquired immune deficiency syndrome (AIDS) [1]. In 2010, there were an estimated 8.8 million incident cases of tuberculosis globally [2]. HIV infection is an important risk factor for tuberculosis, and HIV-infected individuals have a 20 times greater risk of developing tuberculosis as compared to HIV-uninfected individuals [3]. In South Africa, there were an estimated 390,000 incident cases of tuberculosis in 2011 and 65% were co-infected with HIV [1].

Similarly, despite the availability of effective vaccines, influenza virus infection causes substantial annual morbidity and mortality worldwide and in South Africa [4-6]. Globally, it is estimated that influenza epidemics result in three to five million cases of severe illness, and about 250,000-500,000 deaths annually [4]. In 2010 an estimated 12 million episodes of severe and 3 million episodes of very severe acute lower respiratory tract infection resulted in hospital admissions in children <5 years worldwide [5]. A number of risk factors are described for severe outcomes among patients with influenza; these include extremes of age, chronic underlying diseases, pregnancy and immune-suppression [6]. HIV-infected individuals have an increased risk of influenza-associated hospitalisations and mortality [7].

While chronic lung diseases are a known risk factor for severe outcome due to influenza infection, there are few published data on the association between influenza and tuberculosis. Review of data from the 1918 influenza pandemic suggests that individuals with tuberculosis had a disproportionately higher mortality than the general population [8,9] and that underlying tuberculosis infection may have contributed to the elevated mortality observed in young adults [8,10]. There is little information about the interaction between seasonal or pandemic influenza and tuberculosis in recent years. In South Africa, 10% of cases that died during the 2009 A(H1N1)pdm09 pandemic were co-infected with tuberculosis [11]. In South Korea, concurrent tuberculosis and influenza A(H1N1)pdm09 infection was reported in 7 immunocompetent patients, none resulting in fatal outcome [12].

We describe the epidemiological and clinical characteristics of patients admitted with laboratory-confirmed tuberculosis, influenza and tuberculosis-influenza co-infection among patients hospitalized with severe respiratory illness (SRI) in South Africa, a country with a high prevalence of HIV.

## Methods

### Study design

We conducted prospective hospital-based active surveillance for Severe Acute Respiratory Illness (SARI) surveillance at 6 public hospitals in 4 surveillance sites situated across the country: Chris Hani Baragwanath Academic Hospital (CHBAH), Gauteng Province; Edendale Hospital, KwaZulu-Natal Province; the two hospitals of the Klerksdorp–Tshepong Hospital Complex (KTHC), Northwest Province; and Mapulaneng and Matikwana Hospitals, Mpumalanga Province [13]. At each sentinel hospital trained surveillance officers screened all admitted patients for surveillance inclusion criteria from Monday to Friday, except at CHBAH where adult patients were screened on 2 of every 5 days per week because of large patient numbers.

### Case definitions

A case of SARI was defined as a hospitalised individual with onset of illness within seven days of admission meeting age-specific clinical inclusion criteria. We included children aged two days through <3 months with physician-diagnosed sepsis or acute lower respiratory tract infection (ALRI), children aged three months through <5 years with physician-diagnosed ALRI (including, for example bronchitis, bronchiolitis, pneumonia and pleural effusion) and patients aged ≥5 years meeting a modified World Health Organization (WHO) case definition for SARI including sudden onset of fever (>38°C) or reported fever, cough or sore throat, and shortness of breath or difficulty breathing [14]. In addition expanded case definitions were applied at three of the hospitals in two of the surveillance sites, (Edendale Hospital and KTHC) to include individuals meeting the above case definition but with symptoms for >7 days and individuals with suspected or confirmed tuberculosis; these patients were defined as having severe chronic respiratory illness (SCRI). For this analysis severe respiratory illness (SRI) was used to refer to all enrolled patients i.e. patients admitted with an acute or chronic respiratory illness.

The South African National Department of Health tuberculosis case definitions were modified to define suspected tuberculosis [15]. A suspected tuberculosis case in children <12 years was defined as: i) any child presenting with a history of exposure to an infectious tuberculosis case or with a positive tuberculin skin test and symptoms of tuberculosis (chronic cough, weight loss and fever) with or without an abnormal chest x-ray suggestive of tuberculosis, or ii) admitted with a physician diagnosis of tuberculosis or suspected tuberculosis, or iii) initiated on tuberculosis treatment on admission; or iv) was on tuberculosis treatment for <2 months at the time of admission. A suspected tuberculosis case in individuals ≥12 years of age was defined as: i) any patient presenting with night sweats, chronic cough, weight loss or haemoptysis lasting for >2 weeks, or ii) had a physician diagnosis of tuberculosis or suspected tuberculosis, or iii) was initiated on tuberculosis treatment on admission, or iv) was on tuberculosis treatment for <2 months at the time of admission.

Patients who were on tuberculosis treatment for ≥2 months at the time of admission were not enrolled into the surveillance programme as cases of suspected or confirmed tuberculosis (because we assumed that after two months of treatment tuberculosis was no longer the reason for their clinical presentation). These patients were, however, enrolled if they met the criteria for SARI. The tuberculosis status of the enrolled patients was based on results of tuberculosis testing at current admission.

For this analysis, a laboratory-confirmed tuberculosis case was defined as an individual with a positive result for *Mycobacterium tuberculosis* on microscopy, culture or polymerase chain reaction (PCR) by GeneXpert MTB/RIF test (Cepheid, Sunnyvale, California) from the current hospital admission or from a specimen taken within two weeks preceding or following the admission. An influenza case was defined as an individual with a positive PCR test for influenza. An influenza-tuberculosis co-infection case met criteria for both laboratory-confirmed tuberculosis and influenza during the same admission.

### Data collection

A standardized questionnaire was used to collect demographic and clinical data, medical history of the patient and in-hospital outcome. Hospital and intensive care unit (ICU) admission and collection of specimens for bacterial culture, tuberculosis testing and CD4+ T-cell counts were performed according to attending-physician discretion.

### Sample collection and processing

Respiratory specimens (oropharyngeal and nasopharyngeal swabs for patients ≥5 years of age or nasopharyngeal aspirates for children <5 years of age), were collected and placed in 4 ml virus transport medium. Whole blood samples were collected in EDTA-containing vacutainer tubes within 24 hours of hospital admission for the detection of *Streptococcus pneumoniae* and HIV infection.

After collection, respiratory and blood samples were kept at 4°C at the sentinel site, and transported on ice at least twice per week to the National Institute for Communicable Diseases of the National Health Laboratory Services (NICD-NHLS) for testing.

### Detection of respiratory viruses and *S. pneumoniae*

Respiratory samples were tested by multiplex real-time reverse-transcription polymerase chain reaction (PCR) (rRT-PCR) for influenza type A and B, adenovirus, enterovirus, rhinovirus, human metapneumovirus, respiratory syncytial virus and parainfluenza virus types 1–3 [16]. The influenza A positive specimens were subtyped using the Centers for Disease Control and Prevention (CDC) rRT-PCR protocol for detection and characterization of influenza. DNA was extracted from whole blood specimens using the MagNA Pure LC 2.0 instrument and DNA Isolation kit III for bacteria (Roche, Mannheim, Germany) and were tested for the *S. pneumoniae lytA* gene by a quantitative real-time PCR [17].

### Determination of HIV infection

HIV status data were obtained from two data sources. Some patients had HIV testing requested by admitting physicians as part of clinical care. This included HIV enzyme-linked immunosorbent assay (ELISA) testing with confirmation by ELISA on a second specimen for patients ≥18 months of age and qualitative HIV PCR testing for confirmation of HIV-infection status in children <18 months of age. In addition, for consenting patients, linked anonymous HIV PCR testing for children <18 months of age or ELISA for patients ≥18 months of age was performed using a dried blood spot or whole blood specimen at the NICD-NHLS laboratory.

### Determination of tuberculosis infection

Testing for *M. tuberculosi*s, including microscopy, culture, GeneXpert MTB/RIF test, or a combination of these, was undertaken at the discretion of the attending-physician and performed at the laboratory serving the hospital where the patient presented. Specimens for tuberculosis testing were examined by light microscopy for the presence of acid fast bacilli. Tuberculosis culture was performed using the BACTEC MGIT automated culture system (Becton Dickinson, Franklin Lakes, New Jersey). Tuberculosis PCR was performed using the Xpert MTB/RIF system (Cepheid, Sunnyvale, California). The GeneXpert MTB/RIF is an automated sample processing and nucleic acid amplification test for detection of *M. tuberculosis* and is able to detect resistance to rifampicin. The National Department of Health tuberculosis treatment guidelines recommended treatment for patients with positive smears and that patients suspected of tuberculosis should have two specimens tested on microscopy and if these were negative then a 3^rd^ sample should be tested for smear and culture [15].

### Data analysis

To identify factors associated with tuberculosis testing, tuberculosis positivity and tuberculosis-influenza co-infection we included both potential determinants for, as well as outcomes or characteristics of the primary endpoints of the analysis. Univariate comparisons were performed using logistic or multinomial regression. In addition, we implemented three multivariable models to identify factors associated with: (i) tuberculosis testing among enrolled patients; (ii) tuberculosis positivity among enrolled patients tested for tuberculosis; and (iii) tuberculosis single infection compared to influenza single infection or tuberculosis-influenza co-infection. The tuberculosis testing and positivity models were implemented using stepwise forward selection logistic regression. Multinomial regression was used for the comparison of tuberculosis only, influenza only and tuberculosis-influenza co-infected groups and this analysis was performed separately in individuals with symptoms for <7 days and ≥7 days. Multinomial regression allows modelling of outcome variables with >2 categories and relates the probability of being in category *j* to the probability of being in a baseline category. A complete set of coefficients are estimated for each of the *j* levels being compared with the baseline and the effect of each predictor in the model is measured as relative risk ratio (RRR). For this analysis, we used the tuberculosis single infection group as the baseline category and compared it with the influenza single infection and tuberculosis-influenza co-infection groups and we restricted the analysis to laboratory confirmed cases for both influenza and tuberculosis. The general form of the multivariable multinomial model with dichotomous predictors was as follows:

1 $$ \ln \frac{p\left({Y}_j\right)}{p\left({Y}_1\right)}={\beta}_0^{(j)}+{\beta}_1^{(j)}{X}_1+\dots +{\beta}_n^{(j)}{X}_n+\varepsilon $$

Where *p(Y*_*1*_*)* is the probability of the outcome variable in the base category (tuberculosis single infection) and *p(Y*_*j*_*)* is the probability of the outcome variable in the other categories; *j* represents the two other categories (*j = 2*: influenza single infection; and *j = 3*: tuberculosis-influenza coinfection) that are compared to the base category; ${\beta}_0^{(j)}$ is the model constant for category *j*; ${\beta}_1^{(j)}$ to ${\beta}_n^{(j)}$ are coefficients associated with predictors *X*_*1*_ to *X*_*n*_ in category *j*; and *ε* is the error term.

The model in equation 1 was fitted to any enrolled case with available tuberculosis and influenza results irrespective of duration of symptoms as well as separately among individuals with acute (duration of symptoms <7 days) and chronic (duration of symptoms ≥7 days) clinical presentation to specifically assess the clinical characteristics of individuals within these two groups. In order to allow for direct comparison of the final multivariable models for acute and chronic patients we elected to retain any variable which was significant at 0.05 in either one of these models in the final multivariable model. Covariates with a p-value <0.2 at the univariate analysis were assessed for significance at the multivariable analysis and statistical significance was assessed at p < 0.05 for all models. Statistically significant variables were retained in the multivariable models. HIV status was retained in the multinomial models a priori as this is a potential confounder of the interaction between influenza and tuberculosis. Two-way interactions were assessed by inclusion of product terms for all variables remaining in the final additive models. The statistical analysis was implemented using STATA® version 12 (StataCorp, Texas, USA).

### Ethics

Ethical approval for the study was obtained from the University of the Witwatersrand, Human Research Ethics committee (reference M081042) and the University of KwaZulu-Natal Biomedical Research Ethics committee (reference BF157/08). The United States Centers for Disease Control and Prevention deemed this surveillance not to be research and therefore this project did not need human subjects review.

## Results

### Patient enrolment and testing

**Figure 1:**
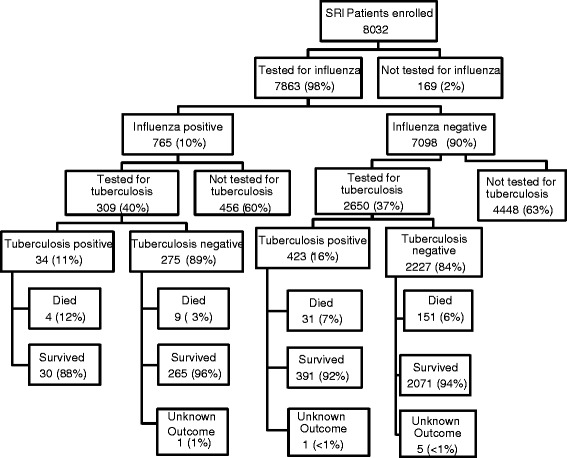
**Flow chart of patients enrolled into the severe respiratory illness (acute and non acute) surveillance, South Africa 2009-2011.**

From June 2010 through December 2011, we enrolled 8032 patients, and influenza results were obtained from 7863 (98%) cases (Figure 1). Of these 4077 (52%) were females and 3291 (42%) were children ≤5 years. Influenza virus was detected in 765 (10%) patients. An influenza subtype was characterized from 764 (99.8%) influenza positive samples, including 219 (29%) influenza A(H1N1)pdm09, 195 (25%) A(H3N2) and 350 (46%) influenza B. Among patients with available influenza results, 2959 (38%) were tested for tuberculosis during admission. Of these, 69% (2029) and 31% (930) met the SARI and SCRI case definitions, respectively. HIV results were available for 2739 (93%) of 2959 patients with available tuberculosis results and 1877 (69%) were HIV-infected. Among influenza tested patients with available data <1% (6/7863) received oseltamivir and <1% (4/7857) were vaccinated against influenza. Two percent (179/7863) of enrolled patients with results for influenza and 2% (49/2959) of those tested for influenza and tuberculosis were already receiving tuberculosis treatment at time of current admission.

### Factors associated with tuberculosis testing on admission

Among patients with available influenza results, on multivariable analysis factors associated with tuberculosis testing were age ≥5 years, with the highest proportion tested (56%, 1378/2473) in the 25–44 age group (adjusted odds ratio (aOR) 3.5, 95% CI 3.0-4.2) as compared to age <5 years, HIV infection (aOR 1.5, 95% CI 1.3- 1.7), symptom duration ≥7 days (aOR 1.3, 95% CI 1.1-1.5), hospitalisation duration >7 days (aOR 1.3, 95% CI 1.1- 1.4), requiring oxygen therapy (aOR 1.2, 95% CI 1.0-1.3) and antibiotic (aOR 1.9, 95% CI 1.4-2.5) or tuberculosis (aOR 1.2, 95% CI 1.0-1.4) treatment started during current admission (Table 1). The proportion of patients tested for tuberculosis was similar for patients testing influenza-positive and influenza-negative and for those who died in-hospital and those who survived.

**Table 1:** **Demographic and clinical characteristics of patients admitted with severe respiratory illness (acute and chronic), by tuberculosis testing status at four sites in South Africa, June 2010- December 2011**

**Characteristic**		**Tested for tuberculosis n/N (%)**	**Unadjusted odds ratio (95% CI)**	**P value**	**Adjusted odds ratios (95% CI)**	**P value**
Age group (years)	0-4	571/3291 (17)	Reference		Reference	
	5-24	270 /587 (46)	4.05 (3.4-4.9)	<0.001	3.3 (2.6-4.1)	<0.001
	25-44	1378/2473 (56)	5.99 (5.3-6.8)	<0.001	3.5 (3.0-4.2)	<0.001
	45-64	611/1197 (51)	4.96 (4.3-5.7)	<0.001	3.2 (2.7-3.8)	<0.001
	≥65	129/315 (39)	3.30 (2.6-4.2)	<0.001	2.6 (2.0-3.4)	<0.001
Sex	Female	1664/4077 (41)	Reference			
	Male	1295/3786 (34)	0.8 (0.7-0.8)	<0.001		
Site	Chris Hani Baragwanath	1618/4437 (36)	Reference		Reference	
	Mapulaneng & Matikwana	144/749 (19)	0.4 (0.3-0.5)	<0.001	0.4 (0.3-0.5)	<0.001
	Edendale	385/1156 (33)	0.9 (0.8-0.99)	0.046	0.7 (0.6-0.8)	<0.001
	Klerksdorp/Tshepong	812/1521 (53)	1.99 (1.8-2.2)	<0.001	1.1 (0.95-1.3)	0.152
Influenza	Negative	2650/7098 (37)	Reference			
	Positive	309/765 (40)	1.1 (0.9-1.3)	0.097		
HIV status	Negative	862/3410 (25)	Reference		Reference	
	Positive	1877/3523 (53)	3.4 (3.0-3.7)	<0.001	1.5 (1.3-1.7)	<0.001
Underlying medical condition*	No	2652/7041 (38)	Reference			
	Yes	307/821 (37)	0.98 (0.85-1.14)	0.879		
Duration of symptoms prior to admission (days)	<7	1884/5957(32)	Reference		Reference	
	≥7	1012/1765 (57)	2.9 (2.6-3.2)	<0.001	1.3 (1.1-1.5)	0.003
Concurrent invasive bacterial infection**	No	653/1832 (36)	Reference			
	Yes	24/46(52)	1.96 (1.1- 3.5)	0.023		
Pneumococcal	No	2435/6284 (39)	Reference			
Infection***	Yes	214/434 (49)	1.5 (1.3-1.9)	<0.001		
Receiving tuberculosis treatment at time of admission	No	158/292 (54)	Reference	<0.001		
	Yes	49/179 (27)	0.3 (0.2-0.5)			
Started on treatment for tuberculosis	No	2301/6716 (34)	Reference		Reference	
	Yes	647/1108 (58)	2.7 (2.4-3.1)	<0.001	1.2 (1.0-1.4)	0.036
Antibiotics prescribed	No	77/332 (23)	Reference		Reference	
	Yes	2877/7515 (38)	2.1 (1.6-2.7)	<0.001	1.9 (1.4-2.5)	<0.001
Oxygen therapy	No	1869/5145 (36)	Reference			
	Yes	1086/2698 (40)	1.2 (1.1-1.3)	0.001	1.2 (1.0-1.3)	0.018
ICU admission	No	2936/7772 (38)	Reference			
	Yes	15/65 (23)	0.5 (0.3-0.9)	0.017		
Duration of hospitalisation (days)	≤7	1913/5666 (34)	Reference		Reference	
	>7	1029/2143 (48)	2.9 (2.6-3.2)	<0.001	1.2 (1.1-1.4)	0.002
Died	No	2757/7321 (38)	Reference	Reference		
	Yes	195/509 (38)	1.02 (0.9-1.2)	0.769		

HIV – human immunodeficiency virus; ICU– intensive care unit, TB-Tuberculosis.

*Underlying conditions included any of the following: Asthma, other chronic lung disease, chronic heart disease (valvular heart disease, coronary artery disease, or heart failure excluding hypertension), liver disease (cirrhosis or liver failure), renal disease (nephrotic syndrome, chronic renal failure), diabetes mellitus, immunocompromising conditions excluding HIV infection (organ transplant, immunosuppressive therapy, immunoglobulin deficiency, malignancy), neurological disease (cerebrovascular accident, spinal cord injury, seizures, neuromuscular conditions) or pregnancy. Comorbidities were considered absent in cases for which the medical records stated that the patient had no underlying medical condition or when there was no direct reference to that condition.

**Concurrent invasive bacterial infections were defined as a bacterial pathogen isolated from blood, cerebrospinal fluid or another sterile site from a specimen taken within 48 hours of hospitalisation; organisms viewed as likely contaminants were excluded.

***Pneumococcal co-infection (lyt A PCR positive for *Streptococcus pneumoniae* on blood specimen).

Other variables evaluated but not presented in the table because they were not significant on univariate analysis were, Oseltamivir treatment prescribed and smoking and alcohol intake for patients ≥12 years.

### Factors associated with laboratory-diagnosed tuberculosis

Among 2959 patients with available tuberculosis and influenza results, 15% (457/2959) tested positive for tuberculosis on the current admission and 2% (10/457) of these were receiving tuberculosis treatment at time of current admission. The highest percentage testing tuberculosis positive was in the 25–44 years age group (18%, 249/1378) (Table 2). Of the patients testing positive for tuberculosis, 332 (73%) were positive on microscopy only. The tuberculosis detection rate was 10% among those with symptoms for <7 days and 25% among those with symptoms for ≥7 days. Of the 457 laboratory-confirmed tuberculosis cases, 198 (43%) and 259 (57%) met the SARI and SCRI case definitions respectively. On multivariable analysis, patients with laboratory-diagnosed tuberculosis were less likely to be co-infected with *S. pneumoniae* (aOR 0.5, 95% CI 0.3-0.9) and to have received oxygen therapy (aOR 0.7, 95% CI 0.5- 0.9). They were more likely to be admitted at KTHC as compared to CHBAH (aOR 1.9, 95% CI 1.4-2.6) and to be started on tuberculosis treatment at current admission (aOR 4.6, 95% CI 3.5-6.0) (Table 2).

**Table 2:** **Demographic and clinical characteristics associated with laboratory-confirmed tuberculosis among patients admitted with severe respiratory illness (acute and chronic) that were tested for tuberculosis and influenza at four sites in South Africa, June 2010- December 2011**

**Characteristic**		**Laboratory confirmed tuberculosis n/N (%)**	**Unadjusted odds ratio (95% CI)**	**P value**	**Adjusted odds ratios (95% CI)**	**P value**
Age group (years)	0-4	55/571 (10)	Reference		Reference	
	5-24	47/270 (17)	1.97 (1.3-3.0)	0.001	1.36 (0.8-2.3)	0.233
	25-44	249/1378 (18)	2.1 (1.5-2.8)	<0.001	1.37 (0.9-2.06)	0.120
	45-64	90/611 (15)	1.6 (1.1-2.3)	0.008	1.11 (0.7-1.7)	0.628
	65+	16/129 (12)	1.3 (0.7-2.4)	0.348	0.94 (0.5-1.9)	0.872
Site	Chris Hani Baragwanath	141/1618 (9)	Reference		Reference	
	Mapulaneng & Matikwana	23/144 (16)	1.99 (1.2-3.2)	0.005	1.4 (0.8-2.3)	0.228
	Edendale	49/385 (13)	1.5 (1.1-2.2)	0.016	0.9 (0.6-1.4)	0.668
	Klerksdorp/Tshepong	244/812 (30)	4.5 (3.6-5.6)	<0.001	1.9 (1.4-2.6)	<0.001
Influenza status	Negative	423/2650 (16)	Reference			
	Positive	34/309 (11)	0.7 (0.5-0.9)	0.023		
HIV status	Negative	89/862 (10)	Reference			
	Positive	335/1877 (18)	1.9 (1.5-2.4)	<0.001		
Underlying medical condition*	No	426/2652 (16)	Reference			
	Yes	31/307 (10)	0.6 (0.4-0.9)	0.007		
Duration of symptoms prior to admission	<7	190/1884 (10)	Reference			
	≥7	255/1012 (25)	3.0 (2.4-3.7)	<0.001		
Pneumococcal co-infection on PCR**	No	400/2435 (16)	Reference		Reference	
	Yes	18/214 (8)	0.5 (0.3-0.8)	0.003	0.5 (0.3-0.9)	0.016
Oxygen therapy	No	337/1869 (18)	Reference		Reference	
	Yes	120/1086 (11)	0.6 (0.4-0.7)	<0.001	0.7 (0.5- 0.9)	0.007
Receiving TB treatment at time of admission	No	25/158 (16)	Reference			
	Yes	10/49 (20)	1.4 (0.6-3.1)	0.456		
Started on TB treatment	No	205/2301 (9)	Reference		Reference	
	Yes	251/647 (39)	6.5 (5.2-8.0)	<0.001	4.6 (3.5-6.0)	<0.001
Duration of hospitalisation (days)	≤7	279/1913 (14)	Reference			
	>7	174/1029 (17)	1.2 (0.96-1.6)	0.096		
Season	Summer	57/366 (15)	Reference			
	Autumn	78/449 (17)	1.1 (0.8-1.6)	0.492		
	Winter	178/1027 (17)	1.1 (0.8-1.6)	0.441		
	Spring	144/1117 (13)	0.8 (0.6-1.1)	0.194		
Died	No	421/2757 (15)	Reference			
	Yes	35/195 (18)	1.2 (0.8-1.8)	0.318		

HIV – human immunodeficiency virus; ICU– Intensive Care Unit, TB-Tuberculosis, CI – confidence interval.

Analysis includes 2029 individuals with severe acute respiratory illness and 930 with severe chronic respiratory illness.

^*^Underlying conditions included any of the following: Asthma, other chronic lung disease, chronic heart disease (valvular heart disease, coronary artery disease, or heart failure excluding hypertension), liver disease (cirrhosis or liver failure), renal disease (nephrotic syndrome, chronic renal failure), diabetes mellitus, immunocompromising conditions excluding HIV infection (organ transplant, immunosuppressive therapy, immunoglobulin deficiency, malignancy), neurological disease (cerebrovascular accident, spinal cord injury, seizures, neuromuscular conditions) or pregnancy. Comorbidities were considered absent in cases for which the medical records stated that the patient had no underlying medical condition or when there was no direct reference to that condition.

**lyt A PCR positive for *Streptococcus pneumoniae* on blood specimen.

Other variables that were evaluated but not presented in the table because they were not significant on univariate analysis were sex, influenza type, concurrent bacterial infection, antibiotic prescribed on admission, admission to intensive care unit, and smoking and alcohol intake for patients ≥12 years.

HIV results were available for 93% (424/457) of tuberculosis-positive and 92% (2315/2502) of tuberculosis-negative individuals. Of these, 335 (79%) of tuberculosis-positive and 1542 (67%) of tuberculosis-negative individuals were HIV infected. Amongst tuberculosis-positive patients tested for HIV, age-specific HIV prevalence were 36% (15/42), 79% (35/44), 95% (222/234), 65% (58/89), 33% (5/15) in the <5 years, 5–24 years, 25–44 years, 45–64 years and ≥65 years age groups, respectively. Amongst tuberculosis-negative patients tested for HIV, age-specific HIV prevalence were 19% (78/417), 71% (147/206), 89% (966/1081), 65% (325/502) and 24% (26/109) in the <5 years, 5–24 years, 25–44 years, 45–64 years and ≥65 years age groups, respectively.

### Factors associated with tuberculosis and influenza infection

Among the 2959 patients with available tuberculosis and influenza results, 2227 (75%) were negative for both pathogens, 423 (14%) were positive for tuberculosis alone, 275 (9%) were positive for influenza alone and 34 (1%) had influenza and tuberculosis co-infection. Tuberculosis treatment was being received at the time of admission for 2% (n = 10) of patients positive for tuberculosis only and <1% (n = 2) of patients positive for influenza only. None of the patients with influenza and tuberculosis co-infection were on tuberculosis treatment at the time of admission.

On multivariable analysis adjusting for age group, patients infected with influenza only compared to patients with tuberculosis only were less likely to be HIV infected (adjusted relative risk ratio (aRRR) 0.6, 95% CI 0.4-0.9), to present with duration of symptoms ≥7 days (aRRR 0.3, 95% CI 0.2-0.4), to be admitted for longer than 7 days (aRRR 0.6, 95% CI 0.4-0.9) while they were more likely to be started on oxygen therapy (aRRR 1.9, 95% CI 1.3-2.8) (Table 3). Patients co-infected with tuberculosis and influenza compared to patients with tuberculosis only were less likely to present with symptoms ≥7 days (aRRR 0.3, 95% CI 0.1-0.7), but were at increased risk of death (aRRR 3.1, 95% CI 1.1-10.1).

**Table 3:** **Factors associated with influenza infection and tuberculosis-influenza co-infection compared with tuberculosis infection only in patients admitted with severe respiratory illness (acute and chronic) and tested for tuberculosis and influenza at four sites in South Africa, June 2010- December 2011**

**Variables**		**Laboratory confirmed tuberculosis Reference** ^**a**^	**Laboratory confirmed influenza**			**Laboratory confirmed tuberculosis-influenza co-infection**		
			**n/N (%)**	**RRR** ^**b**^	**ARRR** ^**c**^ **(95% CI)**	**n/N (%)**	**RRR** ^**b**^	**ARRR** ^**c**^ **(95% CI)**
Age group (years)	0-4	47/423 (11)	46/275 (10)	Reference	Reference	8/34 (22)	Reference	Reference
	5-24	44/423 (10)	28/275 (11)	0.6 (0.3-1.2)	1.3 (0.6-2.9)	3/34 (9)	0.4 (0.1-1.6)	0.5 (0.1-2.5)
	25-44	231/423 (55)	121/275 (44)	0.5 (0.3-0.8)	1.5 (0.8-2.8)	18/34 (53)	0.5 (0.2-1.2)	0.5 (0.2-1.7)
	45-64	86/423 (20)	57/275 (21)	0.7 (0.4-1.1)	1.3 (0.7-2.5)	4/34 (12)	0.3 (0.1-0.9)	0.3 (0.1-2.5)
	65+	15/423 (3)	23/275 (8)	1.6 (0.7-3.4)	3.9 (1.6-9.8)	1/34 (3)	0.3 (0.04-3.4)	9.53e-07
Sex	Female	247/423 (58)	176/275 (64)	Reference		20/34 (59)	Reference	
	Male	176/176 (42)	99/275 (36)	0.8 (0.6-1.1)		14/34 (41)	1.0 (0.5-1.9)	
Site	Chris Hani Baragwanath	124/423 (29)	169/275 (61)	Reference		17/34 (50)	Reference	
	Mapulaneng & Matikwana	21/423 (5)	11/275 (4)	0.4 (0.2-0.8)		2/34 (6)	0.7 (0.1-3.2)	
	Edendale	48/423 (11)	22/275 (8)	0.3 (0.2-0.6)		1/34 (3)	0.2 (0.01-1.2)	
	Klerksdorp/Tshepong	230/423 (54)	73/275 (27)	0.2 (0.2-0.3)		14/34 (41)	0.4 (0.2-0.9)	
HIV status	Negative	82/392 (21)	99/257 (39)	Reference	Reference	7/32 (22)	Reference	Reference
	Positive	310/392 (79)	158/257 (61	0.4 (0.3-0.6)	0.6 (0.4-0.9)	25/32 (78)	0.9 (0.4-2.3)	1.6 (0.5-4.9)
Underlying medical conditions^d^	No	394/423 (93)	240/275 (87)	Reference		32/34 (94)	Reference	
	Yes	29/423 (7)	35/275 (13)	1.98 (1.2-3.3)		2/34 (6)	0.8 (0.2-3.7)	
Duration of symptoms prior to admission	<7	166/412 (40)	194/275 (71)	Reference	Reference	24/33 (73)	Reference	Reference
	≥7	246/412 (60)	81/275 (29)	0.3 (0.2-0.4)	0.4 (0.2-0.4)	9/33 (27)	0.3 (0.1-0.6)	0.3 (0.1-0.7)
Pneumoccocal co-infection on PCR^e^	No	371/388 (96)	233/246 (95)	Reference	Reference	29/30 (97)		
	Yes	17/388 (4)	13/246 (5)	1.2 (0.6-2.6)		1/30 (3)	0.8 (0.1-5.8)	
Oxygen therapy	No	313/423 (74)	158/275 (57)	Reference	Reference	24/34 (71)	Reference	Reference
	Yes	110/423 (26)	117/275 (43)	2.1 (1.5-2.9)	1.9 (1.3-2.8)	10/34 (29)	1.2 (0.5-2.6)	0.97 (0.4-2.3)
Tuberculosis treatment	No	185/422 (44)	243/274 (89)	Reference		20/34 (59)	Reference	
	Yes	237/422 (56)	31/274 (11)	0.1 (0.1-0.2)		14/34 (41)	0.5 (0.3-1.1)	
Hospital duration (days)	≤	257/420 (61)	198/274 (72)	Reference		22/33 (67)	Reference	Reference
	>7	163/420 (39)	76/274 (28)	0.6 (0.4-0.8)	0.6 (0.4-0.9)	11/33 (33)	0.8 (0.4-1.7)	0.7 (0.3-1.5)
Died	No	391/422 (73)	265/274	Reference	Reference	30/34 (88)	Reference	Reference
	Yes	31/422 (7)	9/274	0.4 (0.2-0.9)	0.5 (0.2-1.1)	4/34 (12)	1.7 (0.6-5.1)	3.2 (1.1-10.0)

^a^Tuberculosis only group used as reference for multinomial model analysis (i.e. comparing influenza only to tuberculosis only and tuberculosis–influenza co-infection to tuberculosis only group).

^b^Unadjusted relative risk ratio (RRR) at multivariable analysis.

^c^Adjusted relative risk ratio (ARRR) at multivariable analysis.

^d^Underlying conditions included any of the following: Asthma, other chronic lung disease, chronic heart disease (valvular heart disease, coronary artery disease, or heart failure excluding hypertension), liver disease (cirrhosis or liver failure), renal disease (nephrotic syndrome, chronic renal failure), diabetes mellitus, immunocompromising conditions excluding HIV infection (organ transplant, immunosuppressive therapy, immunoglobulindeficiency, malignancy), neurological disease (cerebrovascular accident, spinal cord injury, seizures, neuromuscular conditions) or pregnancy. Comorbidities were considered absent in cases for which the medical records stated that the patient had no underlying medical condition or when there was no direct reference to that condition.

^e^ *lyt* A PCR positive for *Streptococcus pneumoniae* on blood specimen.

Of the 720 patients testing positive for influenza and/or tuberculosis, 384 (53%) presented with symptom duration <7 days. Of these, 166 (43%) were positive for tuberculosis alone, 194 (50%) were positive for influenza alone and 24 (6%) had tuberculosis-influenza co-infection. Of the 336/720 (47%) patients testing positive for influenza and/or tuberculosis with symptom duration ≥7 days, 246 (73%) were positive for tuberculosis alone, 81 (24%) were positive for influenza alone and 9 (3%) had tuberculosis-influenza co-infection. The median duration of symptoms was 2 (range 0–6) and 14 (7–195) days in patients presenting with symptoms <7 days and ≥7 days respectively.

On multivariable analysis, among patients presenting with symptoms <7 days, patients with influenza only compared to patients with tuberculosis only were less likely to be admitted for >7 days (aRRR 0.4, 95% CI 0.3-0.7). No differences were found in patients co-infected with tuberculosis and influenza compared to patients with tuberculosis only and there was no increased risk of death in influenza tuberculosis co-infected individuals (aRRR 0.8, 95% CI 0.1-8.6) (Table 4).

**Table 4:** **Factors associated with influenza infection and tuberculosis-influenza co-infection compared with tuberculosis infection only among patients presenting with symptoms duration <7 days admitted with severe respiratory illness and tested for tuberculosis and influenza at four sites in South Africa, June 2010-2011**

**Variables**		**Laboratory confirmed tuberculosis Reference** ^**a**^	**Laboratory confirmed influenza**			**Laboratory confirmed tuberculosis-influenza co-infection**		
			**n/N (%)**	**ARRR** ^**b**^	**P-value**	**n/N (%)**	**ARRR** ^**b**^	**P-value**
Duration of symptoms	<7	100/165 (61)	148/194 (76)	Reference		6/9 (67)	Reference	
	≥7 days	65/165 (39)	46/194 (24)	0.4 (0.3-0.7)	0.001	3/9 (33)	0.5 (0.2-1.5)	0.240
HIV status	Negative	46/148 (31)	74/179 (41)	Reference		5/22 (23)	Reference	
	Positive	102/148 (69)	105/ 179 (59)	0.8 (0.5-1.2)	0.280	17/22 (77)	1.6 (0.5-4.7)	0.404
Oxygen therapy	No	101/166 (61)	108/194 (56)	Reference		17/24 (71)	Reference	
	Yes	65/166 (39)	86/194 (44)	1.4 (0.9-2.3)	0.141	7/24 (29)	0.7 (0.3-2.0)	0.560
Died	No	157/166 (95)	191/194 (98)	0.5(0.2-1.5)		24/24 (96)	Reference	
	Yes	9/166 (5)	3/194 (2)	0.2 (0.04-1.2)	0.081	1/24 (4)	0.98 (0.1-8.6)	0.986

HIV – human immunodeficiency virus.

^a^Tuberculosis only group used as reference for multinomial model analysis (i.e. comparing influenza only to tuberculosis only and tuberculosis–influenza co-infection to tuberculosis only group).

^b^Adjusted relative risk ratio (ARRR) at multivariable analysis.

On multivariable analysis among patients presenting with symptoms ≥7 days, patients with influenza only compared to tuberculosis only were less likely to be HIV-infected (aRRR 0.4, 95% CI 0.2-0.7) and more likely to receive oxygen (aRRR 1.7, 95% CI 1.7-5.4). Patients co-infected with tuberculosis and influenza as compared to patients with tuberculosis only were at increased risk of death (aRRR 5.5, 95% CI 1.2-25.3) (Table 5).

**Table 5:** **Factors associated with influenza infection and tuberculosis-influenza co-infection compared with tuberculosis infection only among patients admitted with severe respiratory illness presenting with symptoms duration ≥7 days and tested for tuberculosis and influenza at four sites in South Africa, June 2010- December 2011**

**Variables**		**Laboratory confirmed tuberculosis Reference** ^**a**^	**Laboratory confirmed influenza**			**Laboratory confirmed tuberculosis-influenza co-infection**		
			**n/N (%)**	**ARRR** ^**b**^	**P-value**	**n/N (%)**	**ARRR** ^**b**^	**P-value**
Hospital duration	<7	150/244 (61)	50/80 (62.5)	Reference		6/9 (67)	Reference	
	≥7 days	94/244 (39)	30/80 (37.7)	0.99 (0.6-1.7)	0.991	3/9 (33)	0.6 (0.1-2.7)	0.520
HIV status	Negative	34/233 (83)	25/78 (32)	Reference		1/9 (11)	Reference	
	Positive	199/233 (17)	53/78 (68)	0.4 (0.2-0.7)	0.002	8/9 (89)	2.2 (0.2-20.5)	0.486
Oxygen therapy	No	203/246 (83)	50/81 (62)	Reference		6/9 (67)	Reference	
	Yes	43/246 (17)	31/81 (38)	3.0 (1.7-5.5)	<0.001	3/9 (33)	2.1 (0.5-9.3)	0.315
Died	No	224/245 (91)	74/80 (92.5)	Reference		6/9 (67)	Reference	
	Yes	21/245 (9)	6/80 (7.5)	0.54 (0.2-1.5)	0.875	3/9 (33)	5.48 (1.2-25.4)	0.029

HIV – human immunodeficiency virus.

^a^Tuberculosis only group used as reference for multinomial model analysis (i.e. comparing influenza only to tuberculosis only and tuberculosis–influenza co-infection to tuberculosis only group).

^b^Adjusted relative risk ratio (ARRR) at multivariable analysis.

## Discussion

We describe an increased risk of death associated with influenza co-infection among patients with symptoms ≥7 days hospitalized with laboratory-confirmed tuberculosis in South Africa. Mortality was not increased in patients co-infected with influenza and tuberculosis presenting with a more recent symptom onset. This suggests that influenza infection may contribute to a proportion of the mortality burden amongst patients with tuberculosis, in particular amongst those with a longer duration of symptoms. Because influenza illness is considered to be an acute infection it is possible that it is not considered as a possible diagnosis in patients with longer duration of symptoms. Both influenza and tuberculosis should be considered and tested for among patients hospitalized with pneumonia in settings with high tuberculosis prevalence such as South Africa. Based on the findings of this study our surveillance programme has been amended at two sites to include systematic testing for tuberculosis in patients admitted with severe respiratory illness. In addition, we suggest that where possible tuberculosis testing should be included in SARI programmes in Sub-Saharan Africa where HIV prevalence is high.

A recent study from Thailand did not identify an increased risk of severe outcomes or mortality in patients co-infected with tuberculosis and seasonal influenza when compared to tuberculosis and influenza single infection [18]. The number of patients co-infected with influenza and tuberculosis in this study were, however, small, and patients with a more chronic presentation were not enrolled in the surveillance system. Similar to the findings of our study, ecological and record review studies have reported elevated mortality among tuberculosis-infected individuals during previous influenza pandemics [9,10,19] and 10% of A(H1N1)pdm09 laboratory-confirmed deaths in South Africa in 2009, were co-infected with tuberculosis [11].

In patients presenting with longer duration of symptoms, tuberculosis may have caused lung damage leading to reduced lung capacity and thus impairing the ability to cope with viral infections such as influenza and potentially increasing the severity of illness associated with influenza virus infection. In addition, influenza infection impacts on antibacterial host defenses by either increased production of pro-inflammatory cytokines, causing cell damage [20], or due to inadequate pro-inflammatory chemokine response [21,22], which could increase susceptibility to tuberculosis. Lastly, immunosuppression induced by influenza virus through production of glucocorticoids [23], production of immunosuppressive cytokines [20] or, suppression of T-cell immunity [24] could trigger reactivation of latent tuberculosis and aggravate pulmonary tuberculosis.

Persons with AIDS experience a significant excess mortality due to influenza infection as compared to HIV-uninfected individuals [7,25]. The relative risk of *M. tuberculosis* infection among HIV-infected compared to -uninfected persons varies between 21–37 depending on the country’s HIV prevalence [2]. In our study, a high proportion of patients with tuberculosis were also HIV-infected; however the association with increased mortality in patients co-infected with tuberculosis and influenza virus remained statistically significant even when controlling for concurrent HIV infection in the model.

Despite the high prevalence of tuberculosis in South Africa, in our study, only 37% (3018/8032) of patients admitted with SRI were tested for tuberculosis. Even amongst patients who were initiated on tuberculosis treatment during the current admission, only 58% (656/1123) were tested before initiation of treatment. A study conducted in South Africa reported tuberculosis as the most common cause (40%) of community acquired pneumonia in both HIV-infected and-uninfected patients [26]. A post mortem study in patients dying in hospital found that among patients not suspected of having tuberculosis at time of death, 42% (40/96) were culture positive for tuberculosis and that pneumonia, was the leading admission diagnosis (25%) in those not suspected of tuberculosis [27]. Only 32% of patients with symptom duration of <7 days were tested for tuberculosis, as compared to 57% in those with a chronic presentation, but amongst those tested, 10% had laboratory confirmed-tuberculosis. National guidelines for tuberculosis management advise screening for tuberculosis in patients presenting with persistent chronic symptoms (cough or fever >14 days) [28], however results from a pooled analysis of sensitivity of symptoms for active pulmonary tuberculosis, showed that in studies from sub-Saharan countries, with high burden of HIV, sensitivity of any cough was higher 62% than that of chronic cough >2 weeks, 49% [29]. Failure to test for tuberculosis could lead to delayed or missed diagnosis of tuberculosis, an increase in nosocomial tuberculosis transmission and missed opportunities to identify drug resistance when testing with the GeneXpert assay. Because of the long duration of tuberculosis treatment and possible associated adverse events, a microbiologic diagnosis of tuberculosis should be sought wherever possible [30]. The majority of tuberculosis-positive patients in our study were aged >5 years. Microbiological confirmation of tuberculosis in children is difficult [14,31-34], because childhood tuberculosis tends to be paucibacillary and smear negative. This difficulty can be further compounded by HIV co-infection.

Less than 1% of patients in our study received influenza antiviral treatment or influenza vaccination despite being recommended in national guidelines [35,36]. The association between influenza and tuberculosis seen in our study may be different in settings where influenza vaccination and antivirals are widely used. In South Africa, while there are recommendations for annual influenza vaccination, tuberculosis is not specifically listed as an underlying co-morbid illness to be targeted for vaccination [37]. Tuberculosis treatment guidelines do not routinely include influenza vaccination as part of management [15]. A randomised controlled study of trivalent inactivated influenza vaccine conducted in HIV-infected adults with CD4+ T cell counts >200 μg/ml in South Africa reported 75% (95% CI 9.2-95.6) efficacy in adults [38]. However, patients with tuberculosis co-infection or low CD4+ T cell counts were not included. Although vaccination remains the most effective method to prevent influenza infection, tuberculosis infection suppresses immune responses and thus vaccine effectiveness may be lower in this patient group. There are no published data on the effectiveness of influenza vaccination in patients with tuberculosis, particularly those co-infected with HIV (79% of individuals in our study).

Our study has limitations that warrant discussion. Firstly, we restricted our analysis to patients with tuberculosis laboratory results at current admission, and our case definition of laboratory-confirmed tuberculosis included smear-positive results. As testing for tuberculosis is not 100% sensitive, especially for extra pulmonary tuberculosis which is common in patients with HIV, we may have misclassified a proportion of tuberculosis-positive individuals as not having tuberculosis; however there is no reason to suspect that sensitivity would differ between influenza-positive and -negative patients. The majority of patients with laboratory-confirmed tuberculosis only had microscopy results and it is possible that some may have been infected with mycobacteria other than tuberculosis in a high HIV prevalence setting such as South Africa. However, current South African guidelines recommend initiation of tuberculosis treatment based on smear microscopy results [11]. Secondly we enrolled acute and chronic cases only at two enhanced sites and we didn’t systematically test all enrolled cases for tuberculosis. The patients tested for tuberculosis differed in several characteristics from those not tested. These differences in enrolment and testing procedures as well as the low proportion of patients tested for tuberculosis may have introduced potential selection bias in the population included in the study. Testing all patients admitted with SRI will improve identification of tuberculosis cases and the ability to detect significant differences between groups. We did not collect data on chest X- ray findings. Availability of data on radiological changes in the lungs could have strengthened the evidence in support of our hypothesis regarding changes in the lungs resulting in severe outcome in patients with underlying tuberculosis co-infected with influenza.

## Conclusion

In conclusion, we observed an increased risk of death among tuberculosis-infected patients co-infected with influenza with a symptom history of ≥7 days. The potential public health impact of influenza vaccination among persons with laboratory-confirmed tuberculosis should be explored.

## Abbreviations

AIDS: Acquired immune deficiency syndrome
ALRI: Acute lower respiratory tract infection
aOR: adjusted odds ratio
aRRR: Adjusted relative risk ratio
CDC: Centers for Disease Control
CHBAH: Chris Hani Baragwanath Academic Hospital
ELISA: Enzyme-linked immunosorbent assay
HIV: Human immunodeficiency virus
KTHC: Klerksdorp Tshepong Hospital Complex
ICU: Intensive care unit
*M. tuberculosis*: *Mycobacterium tuberculosis*
NICD-NHLS: National Institute for Communicable Diseases of the National Health Laboratory Services
PCR: Polymerase chain reaction
RR: Relative risk
RRR: Relative risk ratio
SARI: Severe acute respiratory illness
SCRI: Severe chronic respiratory illness
*S. pneumoniae*: *Streptococcus. pneumoniae*
SRI: Severe respiratory illness
WHO: World Health Organization
